# Positive predictive value of automated database records for diabetic ketoacidosis (DKA) in children and youth exposed to antipsychotic drugs or control medications: a tennessee medicaid study

**DOI:** 10.1186/1471-2288-11-157

**Published:** 2011-11-23

**Authors:** William V Bobo, William O Cooper, Richard A Epstein, Patrick G Arbogast, Jackie Mounsey, Wayne A Ray

**Affiliations:** 1 Department of Psychiatry; Vanderbilt University School of Medicine; Nashville, Tennessee USA; 2 Department of Preventive Medicine; Vanderbilt University School of Medicine; Nashville, Tennessee USA; 3 Department of Pediatrics; Vanderbilt University School of Medicine; Nashville, Tennessee USA; 4 Department of Biostatistics; Vanderbilt University School of Medicine; Nashville, Tennessee USA

## Abstract

**Background:**

Diabetic ketoacidosis (DKA) is a potentially life-threatening complication of treatment with some atypical antipsychotic drugs in children and **youth**. Because drug-associated DKA is rare, large automated health outcomes databases may be a valuable data source for conducting pharmacoepidemiologic studies of DKA associated with exposure to individual antipsychotic drugs. However, no validated computer case definition of DKA exists. We sought to assess the positive predictive value (PPV) of a computer case definition to detect incident cases of DKA, using automated records of Tennessee Medicaid as the data source and medical record confirmation as a "gold standard."

**Methods:**

The computer case definition of DKA was developed from a retrospective cohort study of antipsychotic-related type 2 diabetes mellitus (1996-2007) in Tennessee Medicaid enrollees, aged 6-24 years. Thirty potential cases with any DKA diagnosis (ICD-9 250.1, ICD-10 E1x.1) were identified from inpatient encounter claims. Medical records were reviewed to determine if they met the clinical definition of DKA.

**Results:**

Of 30 potential cases, 27 (90%) were successfully abstracted and adjudicated. Of these, 24 cases were confirmed by medical record review (PPV 88.9%, 95% CI 71.9 to 96.1%). Three non-confirmed cases presented acutely with severe hyperglycemia, but had no evidence of acidosis.

**Conclusions:**

Diabetic ketoacidosis in children and youth can be identified in a computerized Medicaid database using our case definition, which could be useful for automated database studies in which drug-associated DKA is the outcome of interest.

## Background

Diabetic ketoacidosis is a severe and potentially life-threatening complication of both type 1 and type 2 diabetes mellitus. While poor adherence to diabetes treatment is the major precipitant of diabetic ketoacidosis (DKA) [1], exposure to atypical antipsychotic drugs has also been linked with DKA development [2,3]. DKA occurrence among individuals exposed to atypical antipsychotic drugs is 10-times that of the general population [4]. The risk of atypical antipsychotic drug-associated DKA may be even higher in children and youth, who appear to be more susceptible to treatment-emergent weight gain and hyperglycemia than adults [5]. Even so, drug-associated DKA occurs infrequently. Clinical trials and prospective cohort studies are therefore unlikely to have sufficient power for detecting inter-drug differences in DKA occurrence.

Large automated medical encounter databases are therefore a valuable data source for observational studies of DKA risk related to atypical antipsychotic drug use, particularly for relatively under-studied patient sub-groups including children and youth [6]. Such databases include computerized records of prescriptions written by clinicians, filled by patients or caregivers or administered in institutions, which provide objective, detailed, reliable, and relatively low-cost measures of drug exposure [6]. Database records of medical encounters may also allow identification of newly diagnosed cases of DKA; however, these records are also subject to misclassification [6,7]. To conduct studies of DKA associated with atypical antipsychotic drug exposure with automated databases, a reliable definition of incident cases is essential. However, we are unaware of a validated computer case definition of DKA.

We therefore tested a computer case definition of incident DKA designed for use in automated databases and report its validation here using a sample from a retrospective cohort study of antipsychotic drug-associated type 2 diabetes risk in a Medicaid population of children and youth.

## Methods

### Sources of Data

The automated database case definition was based upon information from an in-progress retrospective cohort study of antipsychotics on the risk of type 2 diabetes in children and youth [8]. The study utilizes computerized files from the Tennessee Medicaid Program, including an enrollment file and files recording prescriptions filled at a pharmacy, hospital admissions, outpatient visits, and long-term care residence. Medicaid files are linked with computerized death certificates and with the State Hospital Discharge File, an "all-payers" database of hospital discharges and emergency room visits, which provides information occasionally missing from Medicaid files. These files permitted identification of study populations, tracking of study medication use, classification of subjects according to baseline diabetes risk factors, and ascertainment of potential diabetes and DKA cases.

### Study Cohort

The underlying cohort consisted of children and youth (aged 6-24 years) enrolled in Medicaid from 1/1/1996 through 12/31/2007. Cohort membership required that during the past year there was adequate enrollment and health care utilization to assure availability of data needed for study variables; no evidence of life-threatening illness or institutional residence; no diagnosis of schizophrenia, related psychotic disorder, or other condition for which antipsychotics are the only recommended treatment; no evidence of type 1 or type 2 diabetes; and no evidence of pregnancy or polycystic ovarian syndrome (to reduce endpoint misclassification). Cohort members could not have been in the hospital in the past 30 days because Medicaid files do not include in-hospital medications.

The cohort consisted of recent initiators of antipsychotics or control medications (mood stabilizers, ADHD drugs, antidepressants, benzodiazepines). Recent initiators filled a qualifying prescription for a study drug, had no prescription filled more than 90 days prior to the qualifying prescription, and had at ≥ 365 prior consecutive days with no filled prescription. Follow-up began on the day following the prescription fill and ended with the end of the study, the 25th birthday, loss of enrollment, death, failure to meet study inclusion/exclusion criteria, or 365 days following the last day of current use of a study drug, whichever came first.

### Computer Case Definition for DKA

Potential cases of DKA were identified, which served as the study validation sample. Potential cases were identified from inpatient medical care encounter claims with any diagnosis consistent with DKA (ICD-9 250.1, ICD-10 E1x.1). Outpatient encounter claims were excluded. The index date for DKA events was set as the date of hospital admission, unless there was also a claim for emergency room care for any diabetes diagnosis (ICD-9 250x) on the day prior to admission. In this case, the index date was reset to the date of the emergency department visit. From the initial cohort (N = 203,462), a total of 30 potential DKA cases were identified that met these criteria, which served as the study validation sample.

For potential cases, trained study nurses, masked to drug exposure status, abstracted records of pertinent medical care, redacted to conceal patient identifying information. Study nurses confirmed the demographic information from abstracted records, and recorded any pertinent signs, symptoms, laboratory study results, treatment interventions and clinical diagnoses made during hospitalization.

Abstracted records were independently adjudicated by two investigators, masked to case exposure status. Confirmed cases were those in which both adjudicators agreed that the case met pre-specified diagnostic criteria based on current guidelines [9]. This required a random blood glucose > 250 mg/dL and any of the following: a) blood pH < 7.25 (venous) or 7.30 (arterial or capillary); b) blood bicarbonate < 15 mmol/L; c) discharge diagnosis of DKA (ICD-9 250.1, ICD-10 E1x.1). Cases with no laboratory evidence of acidosis were not considered confirmed cases, even if a discharge diagnosis of DKA was given. Study procedures allowed for the resolution of disagreements between adjudicators by a third reviewer; however, no such disagreement occurred. The study was approved by the Institutional Review Board at Vanderbilt University Medical Center.

### Statistical Methods

The positive predictive value (PPV) of the DKA case definition was calculated with 95% confidence intervals (CI) for binomial proportions using Wilson's formula. Case confirmation from medical record review served as the gold standard. Analyses were conducted using STATA statistical software, version 11.0 (STATA Corporation; College Station, Texas, USA).

## Results

Our validation sample was relatively young in age, predominantly Caucasian, and urban-dwelling (Table 1). As expected, the prevalence of mental health diagnoses was high, particularly attention deficit-hyperactivity disorder and mood disorders.

**Table 1:** Demographic and clinical characteristics of validation study sample

Characteristic^a^	All cases	Adjudicated
N	30	27
Age, years, median (IQR)	11 (7-14)	11 (8-15)
Gender, no. (%) female	14 (46.7)	13 (48.1)
Race, no. (%)		
Caucasian	21 (70.0)	19 (70.4)
African-American	6 (20.0)	6 (22.2)
Other	3 (10.0)	2 (7.4)
Urban dwelling, no. (%)	19 (63.3)	17 (63.0)
General medical diagnoses, no. (%)		
Any	15 (50.0)	14 (51.9)
Cardiovascular	1 (3.3)	1 (3.7)
Endocrine (non-diabetic, non-DKA)	0	0
Injury	5 (16.7)	5 (18.5)
Rheumatologic	2 (6.7)	1 (3.7)
Pregnancy	1 (3.3)	1 (3.7)
Psychiatric diagnoses, no. (%)		
Mood disorder	9 (30.0)	8 (29.6)
Anxiety disorder	3 (10.0)	3 (11.1)
Attention deficit-hyperactivity disorder	16 (53.3)	15 (55.6)
Externalizing/impulse control disorders	6 (20.0)	5 (18.5)
Acute stress/adjustment disorders	5 (16.7)	3 (11.1)
Outpatient visits, median no. (IQR)^b^	3 (1-10)	3 (1-10)
Medications, psychotropic, no. (%)		
Antipsychotic drug	6 (20.0)	6 (22.2)
Antidepressant, SSRI or SNRI	9 (30.0)	9 (33.3)
Antidepressant, tricyclic	3 (10.0)	3 (11.1)
Antidepressant, other	1 (3.3)	1 (3.7)
ADHD (stimulants, atomoxetine)	22 (73.3)	19 (70.4)
Benzodiazepine	4 (13.3)	4 (14.8)
Mood stabilizer (VPA, CBMZ, LAM)	2 (6.7)	2 (7.4)

^a ^Corresponds with the value at the beginning of follow-up (t_0_) in the study cohort, unless otherwise specified.

^b ^Corresponds with the value during the year prior to the beginning of follow-up in the study cohort (e.g., the time interval [t_0_-365 days, t_0_]).

Key: ADHD = attention deficit-hyperactivity disorder, CBMZ = carbamazepine, LAM = lamotrigine, SNRI = serotonin-norepinephrine re-uptake inhibitor (venlafaxine, duloxetine), SSRI = selective serotonin re-uptake inhibitor (fluoxetine, paroxetine, sertraline, citalopram, fluvoxamine, escitalopram), VPA = valproic acid.

Of the 30 potential cases identified from the main cohort, 27 (90%) were abstracted and adjudicated. Reasons for incomplete abstraction included the inability to locate the medical record (n = 2) and refusal of the health care facility to participate (n = 1). The full validation sample and adjudicated cases were similar with respect to clinical and demographic characteristics.

Of the 27 adjudicated cases, 24 were confirmed cases of incident DKA (PPV 88.9%, 95% CI 71.9 to 96.1%) (Table 2). The three non-confirmed cases presented acutely with severe hyperglycemia and ketosis, but had no evidence of acidosis.

**Table 2:** Positive predictive value (PPV) of computer case definition for diabetic ketoacidosis

	PPV (%)^a^	95% Confidence Interval^a^
Overall	88.9	71.9 to 96.1
Stratified analyses		
By psychotropic drug exposure^b^		
Antipsychotic drug	83.3	43.6 to 97.0
Other psychotropic drugs	90.0	71.1 to 97.3
By age strata		
Upper (ages 11-24 years)	100.0	81.6 to 100.0
Lower (age ≤ 10 years)	70.0	39.7 to 89.2
By gender		
Male	100.0	78.5 to 100.0
Female	76.9	49.7 to 91.8
By ADHD diagnosis		
Yes	80.0	54.8 to 93.0
No	100.0	75.8 to 100.0

^a ^The positive predictive value (PPV) of the DKA case definition was calculated with 95% confidence intervals (CI) for binomial proportions using Wilson's formula.

^b ^The cohort consisted of recent initiators of antipsychotics or control medications (mood stabilizers, ADHD drugs, antidepressants, benzodiazepines) who filled a qualifying prescription for a study drug, had no fill more than 90 days prior to the qualifying prescription, and had at ≥ 365 prior consecutive days with no filled prescription.

^c ^The underlying cohort consisted of children and youth (aged 6-24 years).

Key: ADHD = attention deficit-hyperactivity disorder

We performed separate PPV calculations for persons in the validation sample who were exposed to antipsychotic drugs, and for those who were exposed to control medications (Table 2). Five of six recent antipsychotic drug initiator cases were confirmed cases of incident DKA (PPV 83.3%, 95% CI 43.6 to 97.0%). Nineteen of 21 cases from recent initiators of control medications were confirmed incident DKA cases (PPV 90.0%, 95% CI 71.1 to 97.3%).

Exploratory subgroup analyses stratified by age, gender and presence of an attention deficit-hyperactivity disorder (ADHD) diagnosis were conducted (Table 2). All seventeen cases from the upper age stratum (ages 11-24 years) were confirmed incident DKA cases, while 7 of 10 cases from the lower age stratum (ages 10 and below) were so confirmed. Incident DKA was confirmed for all 14 cases among males and for 10 of 13 cases among females; and for 12 of 15 cases among individuals with a diagnosis of ADHD and for all 12 cases among those with no ADHD diagnosis.

## Discussion

This study demonstrates that incident DKA cases may be identified with greater than 88% PPV using a computer case definition based on inpatient medical care encounter claims with a diagnosis code consistent with DKA. While uncommon, the risk of antipsychotic drug-associated DKA is important to quantify because it is life-threatening, and because DKA may be the first manifestation of any metabolic disturbance after antipsychotic drug initiation [10,11]. The risk of treatment-emergent metabolic derangements, including DKA, appears to vary according to specific agent [2,11]. However, five cases of DKA have been linked with aripiprazole [12-16], considered to be among the least metabolically-liable atypical antipsychotics [11].

Our case definition included only inpatient claims, a decision that was based on the high likelihood that a majority of DKA cases would require inpatient or emergency medical care. We did not require primary diagnoses of DKA because the clinical criteria for DKA diagnosis are well-established. Therefore, we did not assume that diagnostic reliability was higher for primary DKA diagnoses than that of secondary diagnoses.

To our knowledge, this is the first attempt to validate a computer case definition for DKA intended for use in pharmacoepidemiological studies of DKA as a study endpoint using automated databases. Automated databases may be the only efficient means of quantifying DKA risk associated with specific drug exposures, given how infrequently it occurs. On the other hand, there are several challenges to conducting health outcomes studies using automated databases. The potential for misclassification bias is among the most serious of these [6]. Most automated databases, including the one used in our study, are made up of medical encounter and other service utilization data are collected for purposes other than research, and the quality of the collected data can vary substantially [7]. Thus, computerized medical encounter records are subject to misclassification due to coding errors or other problems [6,7]. Endpoint misclassification is a particular concern for database studies of medical conditions that are not reliably diagnosed or treated [7]. However, the potential for endpoint misclassification also exists for database studies in which the endpoint of interest is DKA, a condition that may reliably come to medical attention due to its acuity and severity. Misclassification errors can introduce bias that cannot be overcome using data analytic or other techniques [6,7]. Therefore, in addition to improving the efficiency of database studies, a validated computer-based DKA endpoint definition is needed in order to reduce the potential for misclassification bias and improve the validity of study findings.

Our DKA computer case definition was developed and validated in a single sample of children and youth in Tennessee Medicaid who recently initiated treatment with a psychotropic medication. Although our case definition has face validity, it is unclear how well the case definition may perform in more general populations including those with existing diagnoses of diabetes mellitus and in adults. One might expect the PPV of our DKA case definition to increase among those with established diagnoses of diabetes mellitus, a necessary precondition for DKA development. However, for many, DKA may be the first manifestation of diabetes mellitus because of delays in the diagnosis and/or treatment of diabetes [17]. One might also suspect that our case definition may perform more poorly in adults because DKA has classically been regarded as a feature of type 1 (rather than type 2) diabetes [18] and a more common complication of diabetes mellitus in children and youth than in adults [19]. However, more recent epidemiological studies have documented increases in the occurrence of DKA in adults and among patients with type 2 diabetes [20], although the majority of DKA cases occur in the setting of type 1 diabetes [21]. Further investigations of our DKA computer case definition in other settings are needed.

Interpretation of our results should proceed with additional limitations in mind. First, our sample size was small, and we were unable to abstract all records sought. The precision of our PPV estimates was reduced as a result. Second, we were unable to determine the sensitivity of our DKA case definition because we did not seek to identify cases presenting in the absence of an inpatient diagnosis. We believe this is unlikely to occur for moderate-to-severe DKA cases. However, some patients with mild DKA may be discharged without subsequent hospital admission after receiving appropriate treatment in the emergency department [22]. Moreover, determining sensitivity (the proportion of true DKA cases that the case definition identifies as having DKA), would quantify performance of the case definition only for those already known to have DKA (established cases). Our objective was to develop a DKA case definition for use in automated database studies, where suspected (not established) cases would be first identified. Our results suggest that a high proportion of these will be true cases using our definition. Third, it should be emphasized that our case definition, which relies on inpatient ICD diagnosis codes from Tennessee Medicaid medical claims data that may be encoded days or weeks following discharge, is applicable to retrospective studies that use automated databases as a data source. Other data collection approaches should be considered for studies designed to identify cases prospectively. Finally, while the rate of DKA misclassification was low, the PPV was lower for antipsychotic initiators in our study compared with control medication initiators. Our results also suggest that the performance of our case definition may vary somewhat depending on which clinical subgroup is under investigation. Larger samples will be needed to determine whether the performance of our case definition varies according to drug exposure or clinical subgroup of interest.

## Conclusions

Diabetic ketoacidosis in children and youth can be identified in a computerized Medicaid database using our case definition, which could be useful for automated database studies in which drug-associated DKA is the outcome of interest.

## Competing interests

In the past, WVB has received research/grant support from Cephalon, Inc., and served on speaker bureaus for Janssen Pharmaceutica and Pfizer, Inc. The other authors declare that they have no competing interests.

## Authors' contributions

WVB wrote the manuscript and researched data. WOC researched data and reviewed/edited the manuscript. RAE contributed to the discussion and reviewed/edited the manuscript. PGA contributed to the discussion and methods, and reviewed/edited the manuscript. JM researched data. WAR edited the manuscript. All authors read and approved the final manuscript.

## Pre-publication history

The pre-publication history for this paper can be accessed here:

http://www.biomedcentral.com/1471-2288/11/157/prepub
